# Erratum

**DOI:** 10.5935/abc.20150103

**Published:** 2015-08

## December issue of 2014, vol. 103 (6), pages. e73-e80

In the Original Article "Case 6/2014 - A Case of a 61-Year-old Woman with Diastolic Heart Failure", pages e73-e80, by authors Fabio Grunspun Pitta, Natalia Quintella Sangiorgi Olivetti, Diego Simões Peniche, Andrea Maria Dercht, Paulo Sampaio Gutierrez, Luiz Alberto Benvenuti, please be aware that the correct spelling for to Andrea Maria Bercht is Andrea Maria Dercht.

## June 2015 Issue, vol. 104 (6), pages 433-442

In the original article "I Brazilian Registry of Heart Failure - Clinical Aspects, Care Quality and Hospitalization Outcomes", published in the June 2015 of the Arquivos Brasileiros de Cardiologia [Arq Bras Cardiol. 2015; 104(6): 433-442], suffered the following correction:

Correcting the text:

In the analysis of the etiologies by region, patients from the South, Southeast and Northeast showed a predominance of the ischemic etiology (33.6%, 32.6%, and 31.9%, respectively). In patients in the Northern region the hypertensive etiology (37.2%) predominated, while among patients in the Northern region the Chagasic etiology predominated (42.4%) (Table 2).

And considering the following text:

In the analysis of the etiologies by region, patients from the South, Southeast and Northeast showed a predominance of the ischemic etiology (33.6%, 32.6%, and 31.9%, respectively). In patients in the Northern region the hypertensive etiology (37.2%) predominated, while among patients in the west-central region the Chagasic etiology predominated (42.4%) (Table 2).

